# Commentary: Triclabendazole induces pyroptosis by activating caspase-3 to cleave GSDME in breast cancer cells

**DOI:** 10.3389/fphar.2025.1583920

**Published:** 2025-04-30

**Authors:** Jie Guo, Wen-Long Peng, Chen-Guang Li, Yu-Long Wang

**Affiliations:** ^1^ Department of Rehabilitation Medicine, Shenzhen Second People’s Hospital, Shenzhen, China; ^2^ Pain Department of Shenzhen Nanshan People’s Hospital, Shenzhen, China

**Keywords:** MCF-7 cells, gasdermin E, caspase-3, pyroptosis, breast cancer

## 1 Introduction

With a discerning and inquisitive interest, we read the paper “Triclabendazole Induces Pyroptosis by Activating Caspase-3 to Cleave GSDME in Breast Cancer Cells” published in Frontiers in Pharmacology (Yan et al., 2021). In this study, Yan et al. present compelling evidence for the pivotal role of triclabendazole in breast cancer cells. Their findings suggest that triclabendazole induces GSDME-dependent pyroptosis through caspase-3 activation, at least partly by enhancing the ROS/JNK/Bax-mitochondrial apoptotic pathway, thereby offering valuable insights into the potential new application of this existing drug in breast cancer treatment. In general, this is an excellent piece of research. However, there are a few points in the paper that require further discussion and critical examination.

## 2 Results and discussion

GSDME expression levels vary in different cell types and tissues. High levels of GSDME lead to pyroptosis while cells with low levels undergo apoptosis upon chemotherapy treatment (Zhang et al., 2019). GSDME has a caspase-3 cleavable site (_267_DMPD_270_), first discovered in cancer cell lines with strong expression of GSDME (Wang et al., 2017; Jiang et al., 2020). If the chemotherapeutic treatment is a caspase-3 activator, it may trigger caspase-3 to cleave GSDME as an alternative to PARP. This mechanism produces GSDME-NT, which may afterwards trigger pyroptotic necrosis in apoptotic cells and release immunogenic effectors by acting on the plasma membrane (Tong et al., 2022). Several chemotherapy medicines have been demonstrated to become ineffective if GSDME is lost. The DNA methyltransferase inhibitor decitabine has been shown in studies to boost the expression of GSDME and induce pyroptosis in cancer tissues with low expression of GSDME by blocking hypermethylation of the gene’s promoter. Therefore, GSDME can be considered to act as a pivotal protein in the process of switching between apoptosis and pyroptosis.

**FIGURE 1:**
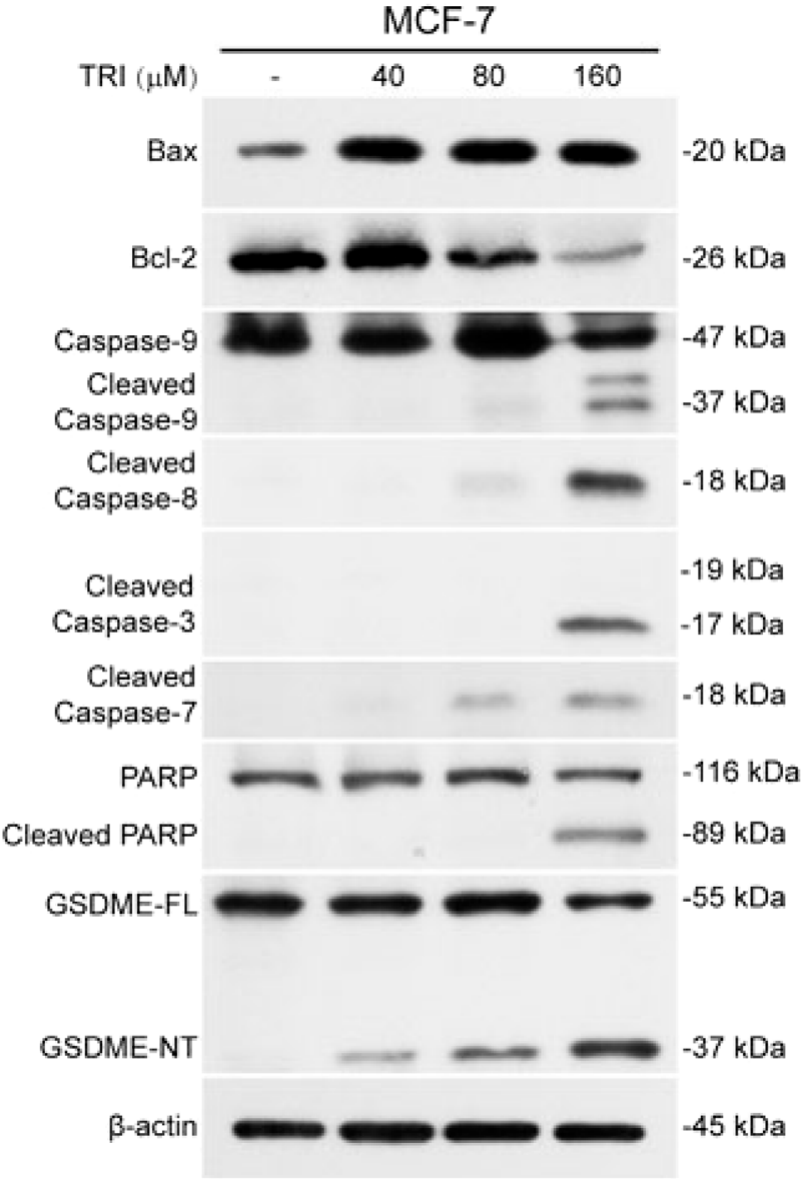
From Yan, et al. “Triclabendazole Induces Pyroptosis by Activating Caspase-3 to Cleave GSDME in Breast Cancer Cells.” Frontiers in pharmacology vol. 12 670081. 8 July 2021 (Yan et al., 2021).

**FIGURE 2:**
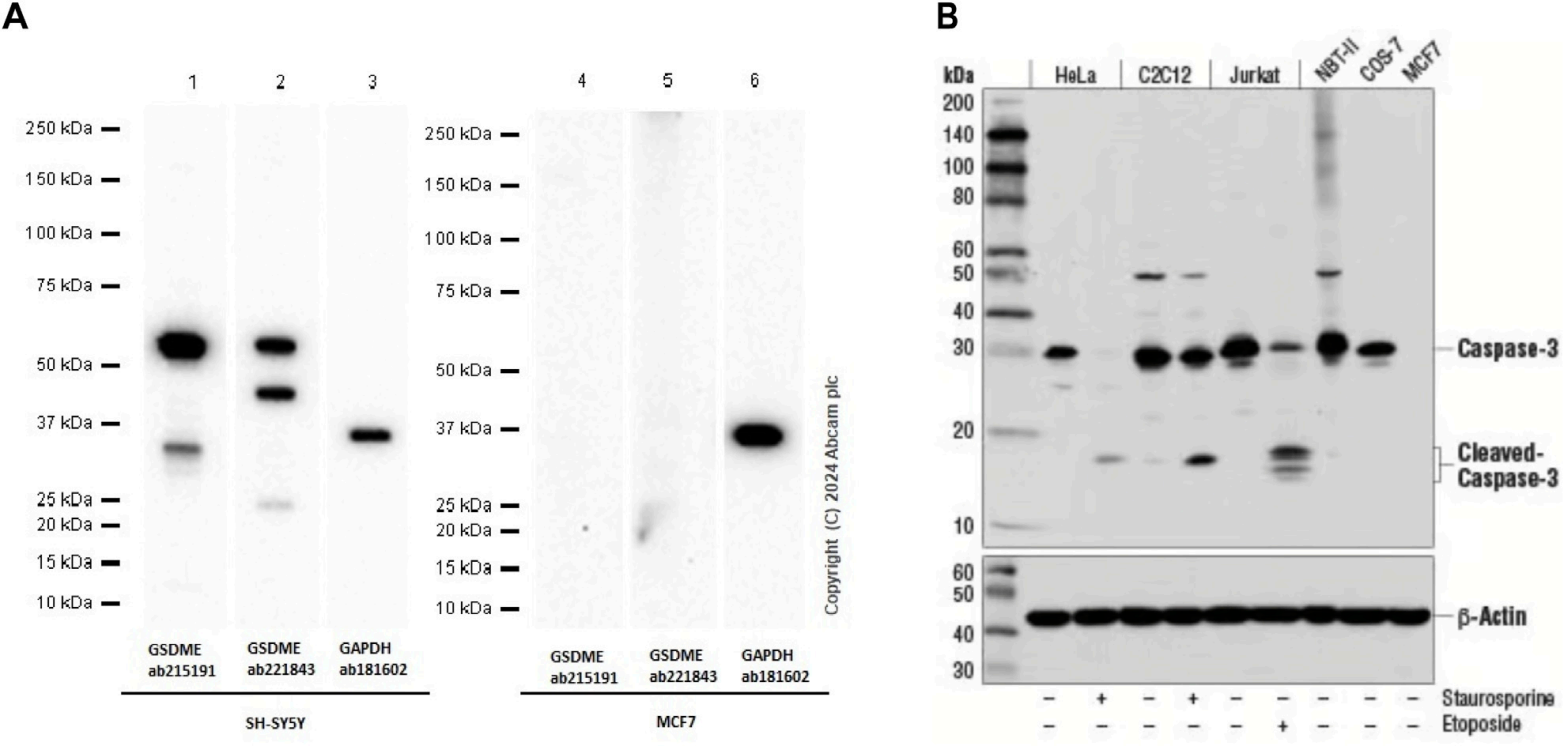
**(A)** Western blot analysis of extracts from SH-SY5Y and MCF-7 cells using GSDME-N-terminal rabbit monoclonal antibody (ab215191). The data were downloaded from the website of Abcam company. **(B)** Western blot analysis of extracts from Hela, C2C12, Jurkat, NBT-II, COS-7 and MCF-7 cells using caspase-3 rabbit monoclonal antibody (#14220). MCF-7 cells are negative for caspase-3 expression. The data were downloaded from the website of Cell Signaling Technology company.

In Yan’s report, the human breast cancer cell line MCF-7 was utilized for *in vitro* experimental analysis. The authors reported that triclabendazole significantly induced the activation of caspase-3, which resulted in an increase in the levels of GSDME-NT in MCF-7 cells (Figure 1). However, it is widely acknowledged that the MCF-7 cells lack the expression of both caspase-3 and GSDME (Tian, 2023). The lack of caspase-3 in MCF-7 cells is caused by a 47-base pair deletion within exon 3 of the *CASP3* gene resulting in the skipping of this exon during pre-mRNA splicing and introduction of a premature stop codon at position 42 that completely abrogates translation of the *CASP3* mRNA. Furthermore, GSDME expression is silenced through promoter hypermethylation, which contributes to the absence of this protein in MCF-7 cells (Janicke et al., 1998b; Friedrich et al., 2001; Ferguson et al., 2003; Tian, 2023). Therefore, the detection of cleaved caspase-3 and GSDME-NT proteins expression in caspase-3/GSDME deficient MCF-7 cells in the present study made us confused. A further apparent problem is that in the section of MATERIALS and METHODS, the primary antibody anti-GSDME-N-terminal (#ab215191), used in the Western blot experiment, is produced by the Abcam company. As illustrated in Figure 2A, the image from the Abcam website depicts a Western blot analysis of extracts from SH-SY5Y and MCF-7 cells utilising GSDME-N-terminal antibody (#ab215191). According to the instructions, anti-GSDME-N-terminal antibody (#ab215191) has explicitly stated that GSDME protein in MCF-7 cells cannot be detected (server as a negative control). Notably, Western blot images provided by Cell Signaling Technology company for the Caspase-3 (D3R6Y) Rabbit mAb (#14220) show that MCF-7 cells are negative for caspase-3 expression (Figure 2B). The central question that needs to be addressed is how cleaved caspase-3 can be detected in the absence of caspase-3 protein expression in MCF-7 cells. Moreover, MCF-7 cells do not express the GSDME protein, thereby precluding the possibility of activated caspase-3 cleaving GSDME. Moreover, caspase-3 plays a crucial role in apoptosis. Although caspase-3-deficient MCF-7 cells are still sensitive to cell death induction by several stimuli including TNF-α, staurosporine and various DNA damaging agents (Janicke et al., 1998a; Janicke et al., 1998b; Janicke et al., 2001). The distinctive morphological characteristics of apoptotic cells, including shrinkage and blebbing, are not evident in caspase-3-deficient MCF-7 cells. It has been demonstrated that exogenous expression of caspase-3 in the MCF-7 cell line can restore the apoptotic characteristics of these cells when stimulated by apoptosis inducers (Janicke et al., 1998b; Yang et al., 2001; Wang et al., 2016).

This discrepancy may be partially attributed to sample contamination and the use of incorrect reagents that cross-react with proteins unrelated to caspase-3 and GSDME in cellular extracts. Another possibility is that the cultures examined may not contain the original MCF-7 cell line, potentially due to contamination with other cell types during the process of culturing and passaging. The authors may need to confirm the identity of the MCF-7 cell line using short tandem repeat (STR) analysis or other appropriate methods. They should also replicate the experiments using a cell line that naturally expresses caspase-3 and GSDME, such as HCT116 or A549, to confirm the proposed mechanism (Yu et al., 2019; Zhang et al., 2019). Additionally, a more reliable approach would be to introduce exogenous expression of caspase-3 and GSDME in MCF-7 cells or use demethylating agents like decitabine to restore GSDME expression.

## 3 Conclusion

In conclusion, Yan et al. have made a valuable contribution to our understanding of the anti-cancer effects of triclabendazole. This work paves the way for new avenues of research and potential treatment strategies in cancer therapy. Although the methodology and results of the study are praiseworthy, there is a need to reinforce the conclusions. Addressing the discrepancy regarding the detection of GSDME-FL, GSDME-NT, and cleaved caspase-3 in the MCF-7 cell line, which is known to lack GSDME and caspase-3 expression, would enhance the reliability of the *in vitro* findings. Furthermore, the validation of pivotal outcomes through the utilisation of exogenous expression of caspase-3 and GSDME, or through the employment of decitabine to induce GSDME expression, may provide more conclusive evidence regarding the impact of triclabendazole on pyroptosis.
